# How to become an excellent pediatric resident: a qualitative comparative study from China

**DOI:** 10.1186/s12913-023-09038-x

**Published:** 2023-01-18

**Authors:** Xingmiao Feng, Yujia Wang, Linjiang Wei, Kai Meng

**Affiliations:** 1 School of Public Health, Capital Medical University, No.10 Xitoutiao, Youanmenwai Street, Fengtai District, Beijing, 100069 China; 2 National Center for Children’s Health, Beijing Children’s Hospital, Capital Medical University, Beijing, China

**Keywords:** Behavioral event interview, Competency, Pediatrics, Qualitative comparative analysis, Standardized training of pediatric residents

## Abstract

**Background:**

Competency-oriented medical education has become a global trend. However, most current studies focus on the independent effects of various competencies and lack an examination of the combined effects. Therefore, the purpose of this study was to explore the competency configurations of excellent pediatric residents and general pediatric residents and to provide a scientific reference for the talent training and career development of pediatric residents.

**Methods:**

Behavioral event interviews were conducted with 23 pediatric residents at a children's hospital in Beijing in July and August 2019. Two researchers coded the interview data to summarize the competency of pediatric residents. The research group scored the performance of 23 pediatric residents in various aspects of competency and used the crisp-set qualitative comparative analysis method to explore the competency configurations of excellent pediatric residents and general pediatric residents.

**Results:**

This study concludes that pediatric residents should have six core competencies: professional spirit, clinical skills, communication ability, learning ability, mental capacity and research ability. There are 4 combinations of competencies for becoming an excellent pediatric resident: the clinical type, scientific research type, all-around development type and high emotional intelligence type. In addition, there are 3 combinations of competencies for becoming a general pediatric resident: the comprehensive ability deficiency type, lack of professionalism and mental capacity type, lack of communication ability type.

**Conclusions:**

There are differences in competence between excellent and general pediatric residents. Excellent pediatric residents do not need to possess all competencies but should specialize in clinical practice, scientific research or communication skills. This study suggests that training in mental capacity, professional spirit and communication ability should be strengthened during pediatric resident training. Pediatric residents should make career development plans according to their actual situation, and hospitals should arrange suitable positions according to the characteristics of pediatric residents.

## Background

Currently, the development of pediatric medical and health service resources in China lags behind the overall level of medical service resources, the supply and demand of pediatric medical and health service systems is seriously unbalanced, and the limited pediatric medical service resources cannot meet the increasing demand for pediatric medical treatment [1, 2]. Some experts note that a shortage of pediatricians is one of the major problems facing China's pharmaceutical industry [3]. Especially since the implementation of China's two-child policy on January 1, 2016, the number of eligible couples has surged, and China's pediatric medical resources must keep pace [4]. By the end of 2021, there were only 151 children's specialized hospitals nationwide and 206,000 pediatric practicing (assistant) physicians, and the number of pediatric practicing (assistant) physicians per 1,000 children was 1.15, which falls short of the standard of 1.5 pediatric practicing (assistant) physicians per 1,000 children in developed countries [5]. In addition, China has a problem with uneven distribution of pediatric medical resources. A lack of education for primary pediatricians and a nonstandard diagnosis and treatment technology process lead to weak service capacity of primary pediatricians that cannot meet the increasing demand for pediatric diagnosis and treatment [6]. The training of pediatricians should not be limited to "quantity" but should also emphasize "quality". Therefore, the training of qualified pediatric medical talent is becoming increasingly urgent.

Standardized training for pediatric residents (STFR) is a crucial link in the process of pediatric talent training. During the training period, theoretical knowledge learned in class can be combined with clinical practice to greatly improve the humanistic qualities, clinical diagnostic skills and treatment levels of pediatric residents in a short time [7]. This is an important part of medical education and an internationally accepted way to improve the diagnosis and treatment abilities of clinicians [8, 9]. Many scholars believe that STFR is the fundamental way to solve the problem of the serious shortage of pediatric talent and its uneven quality. The starting point and foothold of STFR is to train clinicians who meet the standards of competence and can solve practical clinical problems. The concept of competence was first proposed by Professor McClelland in the United States in 1973 [10]. Over decades of continuous development, it has been widely used in many fields. On the basis of “competence”, Professors Epstein and Hundert redefined the concept of "health post competency" as follows: "In daily medical service activities, skillfully use clinical skills, medical knowledge, interpersonal skills, thinking, empathy, and their clinical experience of clinical diagnosis and treatment, etc., to make the service recipient patients benefit" [11]. Competence-oriented education is one of the core contents of the third generation of international medical education reform [12, 13]. Currently, residential training has entered the patient-centered and competency-oriented training stage worldwide. A competency index system or model has been developed for resident doctors, and its rationality and necessity have been fully demonstrated through investigation and practice [14–16]. However, the current assessment system for pediatric residents in China has not established a unified and comprehensive evaluation for assessment and management. The description of the core competency indicators is not comprehensive, and the requirements for humanistic qualities such as professional quality, moral spirit, doctor‒patient communication and teamwork are general and vaguely defined [17]. Especially for pediatrics, due to the particularity of service objects and the complexity of cases and diseases, the corresponding post competency has particular qualities. As a result, it is not possible to conduct a comprehensive and systematic quantitative assessment of the training of Chinese pediatric residents.

Previous studies have mainly analyzed the importance of individual competency factors for pediatricians, such as clinical skills, humanistic literacy, and communication ability [18–20]. However, through interviews with pediatric residents, we found that those who passed the STFR test and obtained a certificate did not have optimal scores in all of these competencies. For example, some pediatric residents have strengths in clinical medical services, while others have strengths in scientific research. The previous single-factor analysis therefore fails to explain the complex mechanism between various competency factors. There is no single optimal path to becoming an excellent pediatric resident; it may involve different combinations of competency factors and other complex mechanisms that encourage pediatric residents to achieve high performance, that is, to become excellent pediatric residents. Moreover, there are differences between the abilities of excellent and general pediatric residents. However, the existing literature lacks empirical studies on how various competency factors influence pediatric residents to produce high performance through complex interactive systems and the differences in competencies between excellent and general pediatric residents. Therefore, we propose the following questions that research in this field has yet to answer: What competencies should pediatric residents possess? Do excellent pediatric residents have to optimize all the competencies required for a residency? How does the difference in competence between pediatric residents affect their ability to become excellent pediatric residents? What is the mechanism by which this occurs? What competencies or configurations of competencies are necessary and sufficient for becoming an excellent pediatric resident? What are the differences in competency between excellent and general pediatric residents? Behavioral event interview (BEI) and qualitative comparative analysis (QCA) methods were used to answer these questions and to understand what competencies pediatric residents should possess, how these competencies work together to develop excellent pediatric residents, and what the differences in competency are between excellent pediatric residents and general pediatric residents. It is important to further improve the effects of resident regulations and training and to help residents make suitable career development plans according to their individual abilities and specialties. At the same time, hospitals can arrange posts according to resident doctors' abilities and specialties to meet their human resources needs.

QCA is a set theory method based on Boolean algebraic logic [21]. Compared with general quantitative statistical analysis methods (such as the linear hypothesis *Y* = *a* + *bX*), QCA can establish a logical connection between causal conditions (or their combination) and outcomes based on the logic of necessity and sufficiency and can further identify synergies between multiple conditional variables rather than isolating the net effect of specific variables [22]. Compared to general qualitative research (such as case studies), QCA ensures that the results can be extrapolated through cross-case analysis [23]. This method is suitable for our study. Since QCA is seldom applied in the health field, this study introduces crisp-set QCA (cs/QCA) and its application steps in detail in the Methods section.

## Methods

### Study sites and sample

In this study, 23 pediatric residents who completed STFR in a children's hospital in Beijing (hereinafter referred to as the "Children's Hospital") in 2017 and 2018 were selected by purposive sampling. Because STFR Chinese pediatric residents was implemented in 2014 and the standardized training time is 3 years, this group of residents in 2017–2018 was the first group of two-year students to complete the training. The results played a guiding role for the subsequent improvement of residential training. The interviews were conducted in July and August 2019. The following conditions were used as the inclusion criteria: (1) completed STFR in the Children's Hospital and passed the completion examination in 2017–2018 and (2) had 1–2 years of clinical work experience in pediatrics after STFR. The following conditions were selected as the exclusion criteria: (1) short-term resident and (2) voluntarily requested to withdraw from the research.

### Behavioral event interviews

#### Interview design and implementation

BEI is one of the classic methods used to construct a competency model [24]. According to the BEI requirements, the interview outline was prepared first. In addition to personal information, work experience and other information about the respondents, the interview asked them to recall their most successful (or most accomplished) and most unsuccessful (or most regrettable) experiences in the course of their clinical practice activities in the previous year and to describe them with the situation, task, action, and result (STAR) rule [25]. Before the formal interviews, the purpose and content of the interviews were explained to the interviewees through the administrative department of the hospital, and informed consent was obtained. After informed consent was obtained, the research group and the interviewee agreed on the time and place of the interview. During the interviews, the researcher asked the interviewees questions according to the interview outline and, given the actual situation, conducted appropriate questioning to obtain as much relevant information as possible. The interviews were audio recorded and recorded in written notes.

#### Data processing and analysis

All interviews were transcribed verbatim into Word documents, and the core competency elements of the pediatric residents were summarized. First, two researchers used NVivo11 software to conduct simultaneous sentence-by-sentence analysis and coding of the original interview data and identified the initial concepts related to the competency of pediatric residents. After the initial concepts were determined, they were further refined and classified, and 15 categories were finally obtained. Second, categories with the same or similar meanings were classified into six main categories. Finally, to ensure reliability and validity, the coding results completed by the two researchers were compared. If there were inconsistencies in the coding results, a third researcher coded them and selected the coding results that were most similar [26]. After the coding was completed, the reliability of the coding was tested by the two indexes of category agreement (CA) and reliability (R). The coding results were deemed to have good reliability when both the CA and R values were greater than or equal to 0.80 [27, 28].

1 $$CA=\frac{2S}{{T}_{1}+{T}_{2}}$$

where T1 represents the number of codes of the first coder, T2 represents the number of codes of the second coder, and S represents the number of the same code.

2 $$R=\frac{2CA}{\left(1+CA\right)}$$

#### The cs/QCA method

QCA, proposed by Charles C. Ragin in the 1980s, includes three main types: cs/QCA, fuzzy-set QCA (fs/QCA) and multi-value QCA (mv/QCA) [29]. For our study, cs/QCA is especially suitable for three fundamental reasons. First, the core competency of pediatric residents is not caused by a single factor; instead, it is the result of the complex interaction of many factors. Unlike traditional statistical methods that focus on the influence of a single independent variable on the outcome, cs/QCA identifies causal conditional patterns that lead to the outcome [22]. Second, in the process of analyzing the configurations that lead to specific outcomes, cs/QCA integrates qualitative and quantitative analysis. Third, the sample size of this study was 23, which is not suitable for analysis by traditional statistical methods, whereas QCA has many advantages for the treatment of small and medium-sized samples.

To aid in the analysis, fs/QCA 3.0 software was used [30]. The software summarized the conditions for encoding information (called a truth table) and used Boolean logic rather than correlation to determine the necessary and sufficient conditions leading to the results as well as the combination of conditions [31]. The software produced three solutions: the complex solution, the parsimonious solution, and the intermediate solution. Referring to the research of Wu et al. and Ageeva et al., the results presented in this study are intermediate solutions [32, 33].

#### Basic steps to apply cs/QCA

Before applying cs/QCA in this study, it was necessary to specify two fit parameters: consistency and coverage. Consistency assesses the degree to which cases that share a condition (or combination of conditions) agree in displaying the outcome, while coverage evaluates the degree to which a condition (or combination of conditions) accounts for instances of the outcome. To understand these parameters, Woodside proposed that in statistical analysis, the former is related to the role of the *P* value in statistical analysis, and the latter is related to R^2^ in linear regression analysis [34].

The basic steps of cs/QCA are summarized as follows. First, the first-level nodes generated by coding were incorporated into fs/QCA 3.0 software to make the dichotomous data table [35]. Second, the necessity of each condition variable was tested, the sufficiency analysis was conducted for the condition variables that could not be used as necessary conditions, and the truth table was constructed [36, 37]. In this study, the consistency threshold was set as 0.80, and the frequency threshold was set as 1 (as the sample was limited in size) [38]. Third, for cases with the same path but different results, appropriate trade-offs were made to solve the contradictory configuration and make the results consistent. Fourth, the results were analyzed. For the QCA results, the path expression method introduced by Fiss and Ragin was used [22]. Based on how the configuration elements are connected to the results, Fiss defines the causal conditions that have a strong causal relationship with the results of interest as core conditions and the factors that have a weak causal relationship with the results as peripheral conditions [39].

### Calibration of causal variables

#### Competency index score

First, based on the interview data, two researchers rated the pediatric residents’ mastery of core competencies. The score of residents who had fully mastered these competencies was 1, and the score of those who had not fully mastered them was 0. Since each competency index could be mentioned several times in an interview, the average score of each competency index of each interviewee was calculated:

3 $$\overline x=\frac{(1\times m+0\times n)}{m+n}$$

where m is the fully mastered frequency and n is the not fully mastered frequency.

#### Generation of dichotomous data table

The competency score of the interviewees was calibrated to obtain the dichotomous data table. Traditionally, QCA assigns each case one of two possible membership scores (1 refers to membership in the set, while 0 refers to nonmembership in the set) based on a crisp-set relationship [40]. In this study, a value below the mean value was set as 0, and a value above the mean value (including) was set as 1. Thus, the dichotomous data table was formed.

### Outcome classification and calibration

According to the results of the annual assessment of the STFR, the participants were divided into excellent performance and general performance groups. In China, the current passing score for STFR is 60 to 80 (the full score is 100), and each province sets its own passing score according to the actual medical situation. Provinces with high-quality medical resources generally set a score cut-off of 80, while provinces with poor medical resources generally set a score cut-off of 60. According to the resident training assessment expert group of the National Talent Exchange Center, a score of 85 is the standard for excellent pediatric residents. Therefore, the research group set the score of 85 as the threshold to classify excellent and general pediatric residents. A score of 85 or above was considered excellent, and a score below 85 was considered general. The code for excellent performance was 1, and the code for general performance was 0.

### Ethics approval

Ethics approval was granted by the Ethics Committee of Capital Medical University (No. Z2019SY055). The study design and information sheets were reviewed by the committee and considered appropriate for use. Participation in the interviews was completely voluntary, and written informed consent was obtained from the participants.

## Results

### Basic interviewee information

There were 23 interviewees, including 9 males and 14 females, who covered 13 departments: endocrinology, respiratory, blood, nutrition, infection, comprehensive, rheumatic immunity, allergic reaction, digestion, neonatal, emergency, nephropathy and the pediatric intensive care unit (Table 1).

**Table 1:** Descriptive statistics of the sample

Categories	Number (*n* = 23)	Percentage (%)
**Gender**		
Male	9	39.13
Female	14	60.87
**Department**		
Endocrinology	1	4.35
Respiratory	3	13.04
Blood	7	30.43
Nutrition	1	4.35
Infection	1	4.35
Comprehensive	1	4.35
Rheumatic immunity	2	8.70
Allergic reaction	1	4.35
Digestion	2	8.70
Neonatal	1	4.35
Emergency	1	4.35
Nephropathy	1	4.35
Pediatric intensive care	1	4.35

### Consistency test and validity analysis of coding results

The coding classification consistency and coding reliability coefficients of the 23 interview texts were all greater than or equal to 0.70. The overall coding classification consistency was 0.79 and the overall coding reliability coefficient was 0.88, indicating that the coding reliability was good (Table 2).

**Table 2:** Coding reliability test

Case ID	T_1_	T_2_	T_1_ + T_2_	S	CA	R
A01	12	14	26	10	0.77	0.87
A02	11	9	20	8	0.80	0.89
A03	12	13	25	10	0.80	0.89
A04	11	15	26	10	0.77	0.87
A05	14	12	26	11	0.85	0.92
A06	12	14	26	10	0.77	0.87
A07	13	11	24	10	0.83	0.91
A08	13	14	27	11	0.81	0.90
A09	9	10	19	7	0.74	0.85
A10	12	13	25	9	0.72	0.84
A11	13	14	27	11	0.81	0.90
A12	12	11	23	10	0.87	0.93
B01	12	10	22	9	0.82	0.90
B02	13	12	25	10	0.80	0.89
B03	13	11	24	9	0.75	0.86
B04	9	11	20	7	0.70	0.82
B05	14	12	26	11	0.85	0.92
B06	9	11	20	8	0.80	0.89
B07	14	12	26	11	0.85	0.92
B08	9	12	21	8	0.76	0.86
B09	13	11	24	10	0.83	0.91
B10	14	10	24	9	0.75	0.86
B11	12	11	23	9	0.78	0.88
**All samples**	276	273	549	218	**0.79**	**0.88**

A refers to general performance; B refers to excellent performance; T1 represents the number of codes of the first coder; T2 represents the number of codes of the second coder; S is the number of the same code; CA represents coding classification consistency; and R represents the coding reliability coefficient

### Coding results

Six main categories of pediatric resident competence were generated: professional spirit, clinical skills, communication ability, learning ability, mental capacity and research ability (Table 3).

**Table 3:** Statistical table of spindle coding and calibration rules

Main categories	Competency concept	Frequency (times)	Mean score	Calibration rules
Professional spirit (PS)	Have a high degree of professional identity and empathy; love medical work; adhere to children as the center; provide children with acceptable, high-quality and comprehensive care; have strong initiative in the pursuit of excellence in medical work	72	0.52	Less than 0.52 was set to 0, and greater than or equal to 0.52 was set to 1
Clinical skills (CS)	Have a certain clinical diagnosis and treatment way of thinking; be able to systematically connect learned knowledge and develop a reasonable diagnosis and treatment plan; master basic clinical operations, together with the entire medical team, to provide patients with comprehensive, high-quality medical services	66	0.66	Less than 0.66 was set to 0, and greater than or equal to 0.66 was set to 1
Communication ability (CA)	Have good interpersonal communication ability; be able to establish a doctor‒patient relationship of mutual trust with patients' parents; have the ability to extract key information to avoid missing important diagnostic information; have the ability to explain the illness and ensure that information about the illness is detailed, comprehensive, and easy to understand	52	0.65	Less than 0.65 was set to 0, and greater than or equal to 0.65 was set to 1
Learning ability (LA)	Have sufficient basic medical knowledge (including clinical medicine, basic medicine, medical ethics and health law knowledge) and the ability of lifelong learning; keep an eye on hot topics and trends in the field; constantly enhance knowledge reserves	39	0.43	Less than 0.43 was set to 0, and greater than or equal to 0.43 was set to 1
Mental capacity (MC)	Have good psychological quality; timely adjustment of mentality under great work pressure in the medical industry; good adaptability and coordination ability when dealing with emergency situations	31	0.12	Less than 0.12 was set to 0, and greater than or equal to 0.12 was set to 1
Research ability (RA)	Have the ability to search and read a large number of studies, to pursue a certain innovation ability and independently write research papers, scientific and technological reports, project applications and other materials	16	0.36	Less than 0.36 was set to 0, and greater than or equal to 0.36 was set to 1

The frequency of a main category is the number of times that it is mentioned in the interview text

### Testing the necessity of a single condition

Ragin suggests that the necessity of a single condition should be tested before exploring the sufficient configurations of conditions for the outcome [22]. To test the necessity of a single condition, we need to specify a consistent threshold. According to the suggestions of Ragin, Schneider and Stroe, 0.9 was used as the threshold [41, 42]. The results indicate that the consistency of the causal condition variables leading to excellent performance results did not reach the critical value of 0.90; hence, there was no necessary condition. Among the causal condition variables leading to general performance results, the consistency of ~ MS was 0.91, making it a necessary condition (Table 4).

**Table 4:** Necessity test of causal conditions

Conditions	Excellent performance results		General performance results	
	**Consistency**	**Coverage**	**Consistency**	**Coverage**
Professional spirit	0.64	0.64	0.36	0.44
~ Professional spirit	0.36	0.33	0.64	0.50
Clinical skills	0.73	0.47	0.73	0.50
~ Clinical skills	0.27	0.50	0.27	0.43
Communication ability	0.73	0.67	0.27	0.27
~ Communication ability	0.27	0.27	0.73	0.67
Learning ability	0.73	0.67	0.27	0.25
~ Learning ability	0.27	0.27	0.73	0.73
Mental capacity	0.27	0.75	0.09	0.25
~ Mental capacity	0.73	0.42	**0.91**	0.53
Research ability	0.55	0.67	0.45	0.45
~ Research ability	0.45	0.36	0.55	0.50

“ ~ ” refers to the negation of conditions

### Analysis of sufficiency

**Fig. 1:**
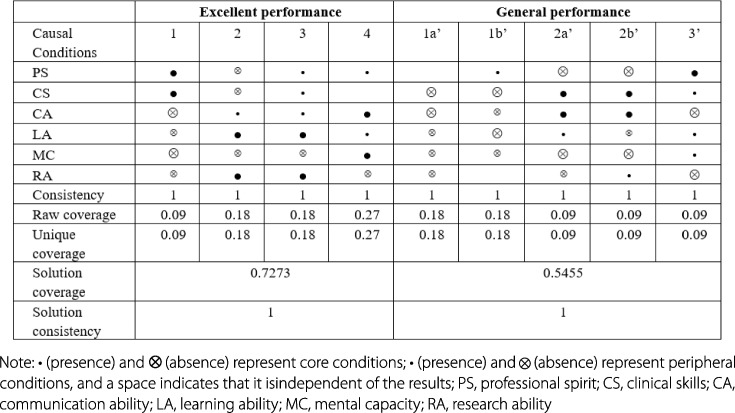
Analysis of sufficient conditions based on QCA

Figure 1 shows that there are four competency configurations that lead to excellent performance results. The coverage of the overall solution is 0.73, indicating that the coverage of the sample is a fairly large share. There are five competency configurations that lead to general performance results, and the overall solution coverage is 0.55. The consistency scores of these conditional configurations are all greater than 0.80, indicating that they are sufficient to justify the results.

In configuration 1, in the presence of professional spirit and clinical skills as the core conditions, despite the absence of communication ability, mental capacity, learning ability and research ability, an individual can become an excellent pediatric resident. This path is called the "clinical type". In configuration 2, with learning ability and research ability combined with good communication ability, even if professional spirit, clinical skills and mental capacity are not present, an individual can become an excellent pediatric resident. This path is called the "scientific research type". In configuration 3, learning ability and research ability exist as core conditions, professional spirit, communication ability and clinical skills exist as peripheral conditions, and only mental capacity does not exist as a peripheral condition. Therefore, this path is called the "all-around development type". In configuration 4, communication ability and mental capacity exist as core conditions, and professional spirit and learning ability exist as peripheral conditions. Despite the absence of scientific research ability, an individual with this configuration can also become an excellent pediatric resident. This path is called the "high emotional intelligence type".

In configurations 1a' and 1b', communication ability and clinical skills do not exist as core conditions, while learning ability, mental capacity and research ability do not exist as peripheral conditions; these two pathways are collectively called the "comprehensive ability deficiency type". In configurations 2a' and 2b', when communication ability and clinical skills exist as core conditions, professional spirit and mental capacity do not exist as core conditions, and learning ability and research ability do not exist as peripheral conditions; individuals with these configurations will become general pediatric residents. This path is called the "lack of professionalism and mental capacity type". In configuration 3', professional spirit exists as a core condition, communication ability and research ability do not exist as core conditions, and the peripheral conditions are learning ability, clinical skills and mental capacity. This path is called the "lack of communication ability type" (Fig. 1).

## Discussion

Based on the BEI method, this study identified six core competencies that pediatric residents should have: professional spirit, clinical skills, communication ability, learning ability, mental capacity and research ability. csQCA was conducted, and a truth table algorithm was used to identify sufficient solutions. The results in Fig. 1 confirm our hypothesis that the core competency of pediatric residents cannot be achieved through a single factor but can be achieved through multiple and equivalent configurations of these causal condition variables. Excellent pediatric residents were mainly the "clinical type", "scientific research type", "all-around development type" and "high emotional intelligence type", while general pediatric residents generally lacked mental capacity.

### Competency index and comparison with existing research

The six competencies identified by this study were consistent with international mature competency models.

### Professional spirit

With the development of medicine, medical education has gradually changed from a focus on natural science knowledge and clinical skills to a focus on patients’ needs [43]. With the proposal of residents’ post competency, residents’ professionalism and humanistic quality are among the six core competencies necessary for resident doctors [44]. Foreign scholars studied occupational spirit in the medical field relatively early, and the United States formed a relatively complete system of physicians’ occupational spirit education and evaluation [45]. The competency model developed by the Accreditation Council for Graduate Medical Education (ACGME) and the American Academy of Medical Sciences after years of research and practice also indicates that residents must possess professional spirit after participating in residential training [46]. The 'Global Standards in Basic Medical Education' created by the World Federation of Medical Education (WFME) state that residents should be able to handle health issues with appropriate and effective empathy and have strong professional ethics [47]. Many studies have shown that professional spirit is an important lever to improve the quality of medical services, so it is important to cultivate doctors’ professional spirit [48]. Many scholars put clinical skills and medical services in first place among the core competencies of clinicians, but the coding results of this study showed that the most frequent occurrence was professional spirit [15]. The results of this study indicate that in their particular specialty, pediatricians are faced with double pressure from children and their families and need to have more empathy and responsibility than clinical skills.

### Clinical skills

Pediatric residents should have a certain type of thinking regarding clinical diagnosis and treatment and should be able to systematically connect the knowledge they have learned to make reasonable diagnosis and treatment plans. They should master basic clinical operations together with the entire medical team to provide patients with comprehensive, high-quality medical services. In the “Administrative Measures for Standardized Training of Resident Doctors (Trial)”, China clearly stated that STFR should train clinicians in solid medical theoretical knowledge and clinical skills for medical institutions at all levels so that they can independently and normatively undertake the diagnosis and treatment of common and multiple diseases in their specialty [49]. The General Medical Council of Britain (GMC) completed a new round of revision of Good Medical Practice in 2013, which has been revised into four core aspects: knowledge, skills and performance; safety and quality; communication, partnership and teamwork; and maintaining trust. The first section states that clinicians should provide a good standard of practice and care. The primary responsibility of physicians is to clearly diagnose patients and effectively treat and relieve pain, which is the most important manifestation of the social value of physicians [50].

### Communication ability

It is necessary to pay attention not only to the improvement of clinical skills but also to the cultivation of residents’ communication ability and consciousness [51]. The communication section of the Good Medical Practice implemented by the GMC states that clinicians should listen and respond to patients’ concerns and preferences, give patients the information they want or need in a way they can understand, respect patients’ right to make decisions about their treatment and care, and support patients in caring for themselves to improve and maintain their health. CanMEDS divides the competence of a good clinician into seven roles, including the role of communicator [15]. In 2014, the “Contents and Standards of Standardized Training for Residents (Trial)” released by the former National Health and Family Planning Commission also included professional interpersonal communication and teamwork in the training objectives of resident doctors [52]. Studies have shown that the most important reason for medical complaints or disputes is poor communication between doctors and patients [53]. Therefore, strengthening doctor‒patient communication can prevent and solve some doctor‒patient disputes [54].

### Learning ability

Self-learning ability is one of the core educational objectives of STFR [55]. The ACGME made this clear when designing core competencies for residency training: "Residents must demonstrate the ability to explore patient care processes, evaluate and integrate scientific evidence, and improve patient care based on continuous self-reflection and lifelong learning", namely, practice-based learning and improvement ability [16]. The medical and health industry requires lifelong learning, practice and continuous education [56]. In particular, the medical knowledge system is rapidly updated with changes in the social economy and technology, so lifelong learning ability is crucial to the cultivation of qualified residents [57].

### Mental capacity

Mental capacity means having good psychological quality, being able to make timely adjustments to one’s mentality under great work pressure in the medical industry, and having good adaptability and coordination ability when dealing with emergency situations. Due to the serious shortage of pediatricians, their work tasks are more onerous than those of other clinicians. At the same time, the onset of pediatric diseases is faster and more urgent than that of other diseases, which makes pediatrics more prone to dangerous situations, leading to high risk and high stress [58]. This requires pediatric residents to have the ability to resist pressure and strain on the basis of sufficient medical knowledge and clinical skills. Doctors’ good mental health status is conducive not only to the improvement of their overall health but also to the diagnosis, treatment and rehabilitation of patients [59]. Therefore, it is necessary to cultivate good psychological qualities in residents and reduce the gap between them and clinical experts [14]. However, there are few relevant studies and evaluation methods in China, and the international model for mature resident competency does not cover this dimension.

### Research ability

The development of medicine depends on innovation in medical knowledge and the deepening of medical research. Clinical practice is the foundation of clinical medicine, but scientific research is the future strength. Without scientific research, clinical medicine will stop moving forward [60]. With only experience and no scientific research to guide clinical practice, the development of medical education will involve the traditional teaching of traditional artisans [61]. Pediatric residents should have the ability to retrieve and read a large number of studies, pursue certain innovation abilities, and independently write various materials, such as research papers, scientific and technological reports, and project applications [62]. Some studies have noted that residents' lack of independent time, uncertain course arrangements, heavy clinical workload and lack of financial support limit the development of their scientific research ability, especially in the aspects of paper writing and statistical analysis [63, 64]. Although there is no rigid requirement for the completion of the residency program, pediatric residents should adjust their degree of mastery according to their own energy, independently pursue certain innovation abilities, and be able to find problems and questions from actual cases to enrich the pediatric care system [65].

### Competency configuration analysis of two groups

#### Excellent performance configurations

Each pediatric resident has a different combination of competencies, and the competencies of excellent pediatricians differ from those of general pediatricians. In this study, there were four types of excellent pediatricians: the clinical type, scientific research type, all-around development type and high emotional intelligence type.

The path of the clinical type is as follows: PS*CS* ~ CA* ~ LA* ~ MC* ~ RA. The interviews also indicated that professional spirit and clinical skills often come together, and pediatric residents with these skills feel a strong sense of achievement when they use their medical skills to provide patients with necessary medical services and achieve good outcomes. At the same time, pediatric residents who focus on clinical services tend to invest time and energy in daily medical activities, so the corresponding work pressure is also greater [66]: "It was the most painful day at work I have ever worked, but I felt a sense of accomplishment to have saved a child."

The scientific research path is as follows: ~ PS* ~ CS*CA*LA* ~ MC*RA. Pediatric residents of this type are often able to determine doubtful or difficult points of a disease through communication with patients and their parents in daily medical activities and consult literature and books independently after work, so their scientific research achievements are relatively fruitful. With the implementation of the dual track of the training of medical graduate students and resident doctors, the requirements for the clinical ability of pediatric residents are increasing, but scientific research training is still in a relatively weak position [67]. An interviewee for this study said, "I think both the Department of Infection and the Department of Neurology are very fond of learning. After finding a suitable case, they will look through the literature or summarize by themselves".

The path of the all-around development type is as follows: PS*CS*CA*LA* ~ MC*RA. The interviews indicated that most of the pediatric residents believed that too little time was allocated to research during the training period, and they had to sacrifice their rest time to balance clinical and scientific research: "Basically, I spent the day in the department and the night in the lab, doing some experiments in my rest time." In this regard, many all-around development pediatricians often feel excessive work pressure and have difficulty regulating their emotions: "In addition to clinical work to do well, the department will also divide some tasks to write articles. I often feel powerless and want to resign.” Approximately 18.18% of the excellent pediatric residents were explained by this approach. A comparison of path 1 and path 3 reveals that if a clinical resident can improve his or her communication ability, learning ability and research ability, he or she can transform into the all-around development type. Similarly, a comparison of path 2 and path 3 reveals that if a resident of the scientific research type can improve his or her professional spirit and clinical skills, he or she can transform into an all-around development type.

The path of the high emotional intelligence type is as follows: PS*CS*LA*MC* ~ RA. The interviews indicated that this type often has a high degree of professional spirit. They believe that pediatrics is a sacred profession, and they are willing to deal with children. Therefore, they can relieve their stress even if work pressure is high. This is the only one of the four paths in which mental capacity exists. This path explained approximately 27.27% of the cases, and the proportion was relatively high.

#### General performance configurations

A comparison of paths 2a' and 2b' revealed that although communication ability and clinical skills exist as core conditions, when professional spirit and mental capacity do not exist, excellence cannot be achieved. The interviews indicated that this type of pediatric resident was relatively less empathetic and more self-centered. Therefore, they tended to become bored and lose their work enthusiasm when faced with high work pressure: "The Children's Hospital was too busy; most of the time was spent doing mechanical work. In addition, the relationship between doctors and patients in pediatrics was also very tense, so there was the idea of giving up" and "Especially when I just moved to a new department, I basically had to work overtime until 10 o'clock at night every day, which made me feel tired physically and mentally anxious.” The two paths each explained approximately 9% of the cases.

Path 3' was the type with poor communication skills. The interviews indicated that many of these pediatric residents had poor communication skills although they were skilled in operation, leading to many conflicts with patients’ parents. Pediatrics is one of the clinical departments where disagreements and conflicts are most likely to arise between doctors and patients [68]. Thus, young pediatricians need to strengthen their doctor‒patient communication ability. Many young pediatricians cannot effectively communicate with children and their parents when faced with the diversity of children and their families and cannot participate in the diagnosis and treatment of clinical diseases [69]. Because children are too young to accurately describe their condition, in most cases, doctors need to rely on their own experience for diagnosis. To enhance family members’ trust, doctors need to understand their anxiety, explain patiently, and inform them about situations that may occur so that family members can anticipate them [70]. Poor communication between doctors and patients is one of the main reasons for violent medical injuries. Only when doctors and patients understand and trust each other can disputes between them be reduced [71]. At the same time, active and effective doctor‒patient communication can enable patients to more comprehensively obtain information related to the disease so that a diagnosis and treatment plan can be developed more quickly and accurately, the medical compliance of the families of sick children can be strengthened, and the medical effect can be improved. Therefore, medical colleges and universities should strengthen residents’ training in communication [72].

The current resident competency model does not involve the dimension of mental capacity alone, but the necessity analysis of the causal conditional variables shows that poor mental capacity is a necessary condition for the general performance of pediatric residents, indicating that mental capacity is also crucial to the career development of resident doctors [73]. In recent years, given the tension in doctor‒patient relationships, the improvement of medical work quality and workload demand, the psychological pressure borne by physicians has increased significantly. In particular, the high work intensity, high medical risk and low remuneration in pediatrics not only affect the quality of doctors’ lives as well as the level of clinical diagnosis and treatment but also cause a variety of psychological illnesses [74]. Positive psychological intervention has significant value in the psychological regulation of physicians, as confirmed by clinical studies [75]. However, the popularization and application of such interventions require research data support. In the future, mental capacity education training for pediatric residents should be strengthened.

## Implications

First, the competency factors of pediatric residents in China have regional and professional particularities. Although many scholars have prioritized clinical skills among the core competencies of clinicians, the coding results of this study showed that the most frequent occurrence was professional spirit, indicating that the competencies of pediatric residents in China have regional and professional particularities. It is inappropriate to evaluate the competency level of Chinese pediatric residents by directly applying the international mature physician competency framework. Therefore, on the basis of BEI with pediatric residents, this study summarizes six core competencies of pediatric residents through coding, which is necessary to indicate a clear direction for STFR in China.

Second, this study finds that a single competency factor is not a necessary condition to become an excellent pediatric resident. While previous research confirmed the importance of professional spirit, clinical skills, and communication ability for pediatricians, this study finds that individual competency factors are not necessary to become excellent pediatric residents. There may be substitution among these competencies, and it is necessary to analyze the functions and mechanisms of competency more systematically. For example, Paths 2 and 3 show the importance of learning ability and research ability to become an excellent pediatric resident, but Path 1 shows that under the condition of poor learning ability and research ability, professional spirit and clinical skills work together to produce an excellent pediatric resident.

Third, this study systematically analyzes the complex causal relationship between competence and becoming an excellent pediatric resident from the perspective of configuration. The results show that there are multiple paths to becoming an excellent pediatric resident rather than a single optimal equilibrium. This provides an explanatory mechanism for how competence influences the development of excellent pediatric residents; that is, each competency is interdependent rather than independent, and there may be a variety of equivalent satisfactory equilibrium states for becoming an excellent pediatric resident.

Fourth, this paper systematically analyzes the complex relationship between competence and becoming an excellent pediatric resident by combining BEI with QCA. In the traditional discussion of causality, the method of correlation analysis is used to obtain the conclusion of correlation. However, QCA can take the form of a "combination" to study what kind of competency combinations can produce excellent pediatric residents and whether there is a certain competency element that is a necessary condition, that is, the "stranglehold" element of becoming an excellent pediatric resident. This provides a new way to study the correlation between competency and pediatric residents’ performance.

## Limitations

There are some limitations in this paper that are worthy of further study in the future. First, the samples for this study were all from the Children’s Hospital, so further investigation to verify and expand on the results in other geographical and cultural contexts is needed. Second, the coding results were inevitably affected by subjective factors of the coders. However, the coders were trained before implementation and a coding consistency test was used to verify the coding results, which compensated for the influence of coding subjectivity to a certain extent [76]. Third, due to the availability of data, this paper could only focus on analyzing the static relationship between the competency and performance of pediatric residents. In the future, with the graduation of batches of pediatric residents who have completed STFR and the accumulation of data, researchers can dynamically analyze how the change in the competency level of pediatric residents affects the change in their performance. Finally, the research focus of this study was not to develop a comprehensive and systematic competency index system for pediatric residents, so it was not conducted in strict accordance with the three-level coding of grounded theory, and Delphi expert consultation was not conducted to demonstrate the resulting competency elements. In the future, it is necessary to build a pediatrician competency model suitable for China's national conditions on the basis of this study to achieve a quantitative evaluation of the competency of Chinese pediatricians, which is of great significance to evaluate the effect of STFR.

## Conclusions

After conducting BEI with 23 pediatric residents, two researchers coded the interview documents and concluded that pediatric residents should possess six competencies: professional spirit, clinical skills, communication ability, learning ability, mental capacity and research ability. The QCA results show that, first, a single competency factor is not a necessary condition to become an excellent pediatric resident; the complex roles of each competency factor are required. Second, this study identified four paths to becoming an excellent pediatric resident. Even if pediatric residents are not optimal in all competency factors, they can build job competency through different combinations of other competencies, and the lack of one or more competencies will not prevent an individual from successfully achieving excellence. These configurations are the clinical type, scientific research type, all-around development type and high emotional intelligence type. These different configurations provide a reference for understanding how to achieve excellent performance among pediatric residents with different combinations of competencies. Third, this study identified three paths to becoming a general pediatric resident. A lack of mental capacity is a necessary condition for general performance, and a lack of professional spirit and communication ability is a sufficient condition for pediatric residents with general performance.

Therefore, this study suggests that pediatric residents’ training in mental capacity, professional spirit and communication should be strengthened in the resident training stage. Pediatric residents should follow a career development route according to their own situation, and hospitals should arrange appropriate positions according to the characteristics of residents to help pediatric residents complete the transformation from resident doctors to excellent pediatricians.

## Data Availability

The datasets generated and/or analyzed during the current study are available from the corresponding author upon reasonable request. Email: mengkai@ccmu.edu.cn.
